# Health and Socio-Demographic Factors Affecting Happiness Among Korean Adults with Moderate and Severe Disabilities: A National Survey-Based Study

**DOI:** 10.3390/medicina61040704

**Published:** 2025-04-11

**Authors:** Seungok An, Su-Yeon Roh, Jeonga Kwon

**Affiliations:** 1 Department of Special Physical Education, Yongin University, Yongin 17092, Republic of Korea; skyforve@yiu.ac.kr; 2 Department of Exercise Rehabilitation, Gachon University, Incheon 21936, Republic of Korea; 3 Department of Elementary Education, College of First, Korea National University of Education, Cheongju 28173, Republic of Korea

**Keywords:** adults, disability, happiness, health-related factors, suicide

## Abstract

*Background and Objectives*: This study identified health-related factors that affect the happiness of Korean adults with disabilities based on the disability level. *Materials and Methods*: Data of 7581 adults with disabilities aged 20 or older were collected from the 2023 Survey on the Status of Persons with Disabilities. The collected data were analyzed using frequency and multivariate logistic regression analyses. *Results*: Among individuals with moderate disability, males were less happy than females. Those with elementary school or lower education levels were less happy than those with college or higher education levels. Those who experienced stress, sadness and despair, suicidal thoughts, or discrimination were less happy than those who did not. In contrast, married individuals were happier than unmarried individuals. Those who exercised, went out alone, engaged in paid work, or participated in social activities were happier than those who did not. Among individuals with severe disabilities, males were less happy than females. Those with lower levels of education were less happy than those with higher levels of education. Those who experienced stress, sadness and despair, suicidal thoughts, suicide attempts, difficulty in communication, or discrimination were less happy than those who did not. Those who perceived their body as very thin were less happy than those who perceived it as very obese. Those who suffered from chronic disease were less happy than those who did not. In contrast, those aged 20–29 were happier than those aged 80 years or older. Married individuals were happier than unmarried ones. Those who exercised, went out alone, engaged in paid work, or participated in social activities were happier than those who did not. *Conclusions*: Factors affecting the happiness of Korean individuals with disabilities differ depending on the disability degree. Therefore, health policies, plans, and support measures must be established based on the disability degree to support daily physical activities, along with health services facilitating physical activity.

## 1. Introduction

What is happiness? Happiness is the psychological state of well-being as well as a positive health indicator [1]. According to the Declaration of Independence, all individuals have the right to pursue happiness [2]. This raises the question: Is everyone happy? The meaning of happiness and its extent can differ based on one’s situation, conditions, and environment [3]. In other words, happiness is an important quality-of-life outcome and is an indicator of whether an individual is living in a healthy manner. In particular, because people with disabilities are less healthy than ordinary people, examining their happiness is important not only because it is a measure of their quality of life but also because it is a measure of a healthy society [1,2,3].

Are individuals with disabilities happy? According to anti-discrimination laws and the Convention on the Rights of Persons with Disabilities, there have been improvements in physical environments as well as attitudes toward disability [4]. However, individuals with disabilities are often stigmatized and underprivileged, and they are assumed to be limited in their functional abilities and performance of different roles [5,6]. These individuals live unequal lives and experience stigma, discrimination, and alienation. Furthermore, their happiness and life satisfaction are significantly lower than those of individuals without disabilities [7]. Alternatively, there is also the perspective of the “disability paradox”, suggesting that people with disabilities can be happier and have a higher quality of life than non-disabled people and that this is possible through social support and acceptance [7].

The term persons with disabilities refers to persons who have long-term or recurring physical, mental, sensory, psychiatric, or learning impairments and who consider themselves to be disadvantaged in employment because of that impairment [8]. In other words, disability refers to the lack of an ability to perform activities in a manner or range that is considered normal for a human being [9]. Individuals with disabilities generally have poor physical, mental, and social health due to limited abilities. Often, these aspects affect one another and create a vicious cycle. For example, individuals with physical disabilities have fewer opportunities to participate in social activities, which negatively affects their mental health, as they may feel stressed or even contemplate suicide [10,11,12,13,14,15].

Disability and health are closely related; thus, exploring health-related factors that influence the happiness of individuals with disabilities may help improve their limited abilities, mitigate their problems, and increase their happiness levels. Many studies have examined the health-related factors and happiness levels of individuals with disabilities. For example, one study explored how participation in physical activity affects their happiness levels [16,17]. Another study explored the relationship between social interaction and their happiness levels [18,19].

However, disabilities can differ in terms of their degree, and the health status and happiness levels of individuals with disabilities differ depending on the disability degree [20]. Prior studies were not characterized by the disability degree. Therefore, it is necessary to explore factors that affect happiness according to the disability degree. Consequently, the health-related factors influencing their happiness may also differ depending on the degree of disability. This makes it imperative to identify health-related factors affecting the happiness of individuals with disabilities based on the degree of disability.

Therefore, this study aimed to explore health-related factors that influence the happiness of Korean adults with disabilities based on the disability degree. Such an assessment can enhance not only the health but also the rights of Korean adults with disabilities. Additionally, this assessment can result in the provision of tailored support, thus supporting efforts to build an equal society devoid of discrimination. This study investigated the following research question: what are the health-related factors that influence the happiness of Korean adults with disabilities based on the degree of disability? The results of this study are expected to be used to establish policies that increase the happiness of people with disabilities. Furthermore, these results are expected to be used as a highly relevant basic dataset to aid the development and establishment of policies and health services to improve the quality of life of people with disabilities tailored to different levels of disability.

## 2. Materials and Methods

### 2.1. Design and Data Collection

This study used survey data from the 2023 Survey on the Status of Persons with Disabilities conducted by the Korea Ministry of Health and Welfare. This survey targets individuals with disabilities using two-stage cluster sampling. In the 2023 survey, 8000 registered individuals with disabilities were surveyed at the end of May 2023. The investigators visited the homes of these individuals, and well-trained researchers interviewed them. After excluding the data of 419 individuals aged 19 or younger, this study utilized the data of 7581 individuals with disabilities aged 20 or older. The Korea Ministry of Health and Welfare collects the survey data, converts it into tabular data, deletes personally identifiable information, and publishes the data on its website. We requested access to these data, completed the data use pledge (security memorandum), and submitted our research plan on the Korea Health and Welfare Data Portal. The dataset was used after receiving approval https://data.kihasa.re.kr/kihasa/kor/contents/ContentsList.html (accessed on 5 March 2025). The 2023 Survey on the Status of Persons with Disabilities was approved by the Institutional Review Board of the Korea Ministry of Health and Welfare (approval number: 117032; 31 December 2022) and conducted according to the principles outlined in the Declaration of Helsinki. All participants and their guardians were informed of the study’s purpose, and they voluntarily signed an informed consent form.

### 2.2. Variables

The variables in this study were happiness, demographic factors, and health-related factors. The variables and classification of health-related factors were established on the basis of the questionnaire items. The questionnaire was divided into the following sections: general characteristics, health characteristics, social characteristics, and economic characteristics. The demographic factors were age, sex, disability level, education level, and marital status. The health-related factors comprised physical health factors (perceived body shape, exercise participation, presence of chronic disease, requirement of walking assistance, and ability to go out alone), mental health factors (degree of stress, presence of sadness and despair, presence of suicidal thoughts, engagement in suicide attempts, and experience of psychological counseling), and social health factors (engagement in paid work, difficulty in communication, utilization of health services, degree of discrimination, and participation in social activities). Happiness was assessed by asking respondents, “How happy are you now?”. Respondents rated their happiness from 0 (“unhappy”) to 10 (“happy”). We categorized ratings from 0 to 3 as “unhappy”, 4 to 6 as “neutral”, and 7 to 10 as “happy”. Sex was categorized as male or female. For age, the respondents were asked to directly enter their age. We categorized age as 20–29 years, 30–39 years, 40–49 years, 50–59 years, 60–69 years, 70–79 years, and 80 years or older. The disability level was determined by asking respondents, “What is the level of your disability?”. The response options were “moderate disability” and “severe disability”. The education level was determined by asking, “What is your highest level of education?”. The response options were “preschool”, “unschooled”, “elementary school”, “middle school”, “high school”, “vocational college”, “college (3 years or less)”, “university (4 years or more)”, and “graduate school or higher”. We categorized the responses as “elementary school or below”, “middle school”, “high school”, and “college or higher”. Marital status was assessed by asking respondents, “Are you married?”. The response options were “married”, “widowed”, “divorced”, “separated”, “other (single mother/single father, etc.)”, and “unmarried”. We categorized these responses as “married”, “widowed/divorced/separated/other (single mother/single father, etc.)”, and “unmarried”.

Perceived body shape was assessed by asking respondents, “What do you think your body shape is?”. The response options were “very thin”, “slightly thin”, “normal”, “slightly obese”, and “very obese”. Exercise participation was determined by asking, “In the past year, did you exercise for health management?”. The response options were “yes” and “no”. The presence of chronic disease was determined by asking, “Have you been suffering from a disease for more than three months?”. The response options were “yes” and “no”. The requirement of walking assistance was assessed by asking, “To what extent can you walk on your own?”. The response options were “no assistance required”, “some assistance required”, “considerable assistance required”, and “full assistance required”. The ability to go out alone was determined by asking, “Are you able to go out alone?”. The response options were “yes” and “no”.

The degree of stress was measured by asking respondents, “How much stress do you feel in your daily life?”. The response options were “every moment”, “a lot”, “a little”, and “almost never”. The presence of sadness and despair were assessed by asking, “In the past year, did you feel sad or hopeless for more than two weeks in a row to the point it interfered with your daily life?”. The response options were “yes” and “no”. The presence of suicidal thoughts was determined by asking, “In the past year, did you think about wanting to die?”. The response options were “yes” and “no”. Engagement in suicide attempts was determined by asking, “Did you attempt to commit suicide in the past year?” The response options were “yes” and “no”. The experience of psychological counseling was assessed by asking respondents, “In the past year, did you receive psychological counseling via telephone, Internet, or in-person visit?”. The response options were “yes” and “no”.

Engagement in paid work was assessed by asking respondents, “In the last week, did you work for more than an hour for the purpose of earning money?”. The response options were “yes” and “no”. Difficulty in communication was assessed by asking, “Do you experience difficulty in communication?”. The response options were “yes” and “no”. The utilization of health services was determined by asking, “Did you receive any health care or health-related services in the past year?”. The response options were “yes” and “no”. The degree of discrimination was evaluated by asking, “To what extent do you feel discriminated against because of your disability?”. The response options were “always”, “sometimes”, “rarely”, and “not at all”. Participation in social activities was determined by asking, “Did you participate in social activities (meeting friends and relatives, gatherings, etc.) in the past week?”. The response options were “yes” and “no”. Except for the responses on happiness, age, education level, and marital status, we used respondents’ responses without any modifications. Because this study aimed to explore health factors that affect the happiness of people with disabilities, the covariate variable was not specified.

### 2.3. Data Analysis

We analyzed data in the following manner. First, we conducted a frequency analysis on respondents’ characteristics. Second, multivariate logistic regression analyses were conducted to identify the determinants of the happiness of individuals with disabilities based on their disability level. Logistic regression models were used to model nonlinear relationships between independent and dependent variables. Such models are useful research tools for the identification of trends because they reflect the nonlinear relationships between independent and dependent variables and are able to model complex relationships. Because of their characteristics, logistic regression analyses are often used to explore factors. All statistical analyses were performed using SPSS for Windows (version 23.0; IBM Corp., Armonk, NY, USA), and statistical significance was set at *p* < 0.05.

## 3. Results

### 3.1. Characteristics of the Respondents

Table 1 presents the demographic characteristics of the respondents. Among 7581 respondents, most were male (60.3%) and reported feeling neutral when asked how happy they felt (50.6%). Regarding the disability level, 52.8% of the respondents reported moderate disability, whereas 47.2% reported severe disability. Regarding mental health factors, many respondents reported feeling a little stressed (45.0%). Most reported the absence of sadness and despair (86.3%), suicidal thoughts (90.5%), and suicide attempts (99.2%) and had not received psychological counseling (94.2%). Regarding physical health factors, many respondents believed that they had a normal body shape (47.0%). Most respondents engaged in exercise (65.8%) and were able to go out alone (80.4%). Regarding social health factors, 33.0% of the respondents engaged in paid work, 75.4% did not experience difficulty in communication, 98.5% utilized health services, and 50.4% participated in social activities. Most respondents did not require walking assistance (76.1%). Furthermore, many respondents reported that they rarely felt discriminated against (49.4%).

### 3.2. Factors That Influence the Happiness of Individuals with Moderate Disability

Table 2 presents the results of determining the factors that influence the happiness of individuals with moderate disability. Sex, marital status, degree of stress, presence of sadness and despair, presence of suicidal thoughts, exercise participation, ability to go out alone, engagement in paid work, degree of discrimination, and participation in social activities were associated with feeling neutral among individuals with moderate disability. Those who were male were 0.589 times (95% confidence interval [CI]: 0.444–0.780, *p* < 0.001) less likely to feel neutral than those who were female. Those who were married were 2.014 times (95% CI: 1.266–3.202, *p* = 0.003) more likely to feel neutral than those who were unmarried. Those who felt stressed every moment and those who felt stressed a lot were 0.200 times (95% CI: 0.115–0.348, *p* < 0.001) and 0.368 times (95% CI: 0.237–0.571, *p* < 0.001) less likely to feel neutral than those who almost never felt stressed, respectively. Those who experienced sadness and despair were 0.587 times (95% CI: 0.426–0.809, *p* < 0.001) less likely to feel neutral than those who did not. Those who had suicidal thoughts were 0.555 times (95% CI: 0.392–0.785, *p* < 0.001) less likely to feel neutral than those who did not. Those who exercised were 1.654 times (95% CI: 1.274–2.147, *p* < 0.001) more likely to feel neutral than those who did not. Those who could go out alone were 1.538 times (95% CI: 1.038–2.279, *p* = 0.032) more likely to feel neutral than those who could not. Those who engaged in paid work were 2.121 times (95% CI: 1.507–2.986, *p* < 0.001) more likely to feel neutral than those who did not. Those who sometimes experienced discrimination were 0.636 times (95% CI: 0.421–0.960, *p* = 0.031) less likely to feel neutral than those who did not experience discrimination at all. Those who participated in social activities were 1.349 times (95% CI: 1.035–1.758, *p* = 0.027) more likely to feel neutral than those who did not.

Sex, education level, marital status, degree of stress, presence of sadness and despair, presence of suicidal thoughts, exercise participation, ability to go out alone, engagement in paid work, degree of discrimination, and participation in social activities were associated with feeling happy among individuals with moderate disability. Those who were male were 0.397 times (95% CI: 0.291–0.542, *p* < 0.001) less likely to feel happy than those who were female. Those who had elementary school or lower education levels were 0.364 times (95% CI: 0.231–0.575, *p* < 0.001) less likely to feel happy than those who had college or higher education levels. Those who were married were 6.006 times (95% CI: 3.549–10.162, *p* < 0.001) more likely to feel happy than those who were unmarried. Those who felt stressed every moment, those who felt stressed a lot, and those who felt a little stressed were 0.063 times (95% CI: 0.032–0.122, *p* < 0.001), 0.092 times (95% CI: 0.058–0.148, *p* < 0.001), and 0.395 times (95% CI: 0.252–0.619, *p* < 0.001) less likely to feel happy than those who almost never felt stressed, respectively. Those who experienced sadness and despair were 0.429 times (95% CI: 0.280–0.658, *p* < 0.001) less likely to feel happy than those who did not. Those who had suicidal thoughts were 0.279 times (95% CI: 0.167–0.468, *p* < 0.001) less likely to feel happy than those who did not. Those who exercised were 2.369 times (95% CI: 1.765–3.180, *p* < 0.001) more likely to feel happy than those who did not. Those who could go out alone were 1.826 times (95% CI: 1.116–2.988, *p* = 0.017) more likely to feel happy than those who could not. Those who engaged in paid work were 3.726 times (95% CI: 2.598–5.345, *p* < 0.001) more likely to feel happy than those who did not. Those who always experienced discrimination, those who sometimes experienced discrimination, and those who rarely experienced discrimination were 0.125 times (95% CI: 0.048–0.323, *p* < 0.001), 0.259 times (95% CI: 0.166–0.404, *p* < 0.001), and 0.483 times (95% CI: 0.325–0.718, *p* < 0.001) less likely to feel happy than those who did not experience discrimination at all, respectively. Those who participated in social activities were 2.246 times (95% CI: 1.680–3.002, *p* < 0.001) more likely to feel happy than those who did not.

These results suggested that, among individuals with moderate disability, males are less happy than females. Those with elementary school or lower education levels are less happy than those with college or higher education levels. Those who are married are happier than those who are unmarried. Those who experience stress, sadness and despair, suicidal thoughts, or discrimination are less happy than those who do not. In contrast, those who exercise, go out alone, engage in paid work, or participate in social activities are happier than those who do not.

### 3.3. Health-Related Factors That Influence the Happiness of Individuals with Severe Disability

Table 3 presents the results of determining the factors that influence the happiness of individuals with severe disability. Education level, degree of stress, presence of sadness and despair, engagement in suicide attempts, exercise participation, requirement of walking assistance, ability to go out alone, engagement in paid work, and participation in social activities were associated with feeling neutral among individuals with severe disability. Those who had elementary school or lower education levels were 0.704 times (95% CI: 0.501–0.989, *p* = 0.043) less likely to feel neutral than those who had college or higher education levels. Those who felt stressed every moment and those who felt stressed a lot were 0.296 times (95% CI: 0.194–0.452, *p* < 0.001) and 0.404 times (95% CI: 0.290–0.562, *p* < 0.001) less likely to feel neutral than those who almost never felt stressed, respectively. Those who experienced sadness and despair were 0.560 times (95% CI: 0.425–0.737, *p* < 0.001) less likely to feel neutral than those who did not. Those who engaged in suicide attempts were 0.242 times (95% CI: 0.106–0.551, *p* < 0.001) less likely to feel neutral than those who did not. Those who exercised were 1.974 times (95% CI: 1.572–2.412, *p* < 0.001) more likely to feel neutral than those who did not. Those who required some walking assistance and those who did not require walking assistance were 1.826 times (95% CI: 1.251–2.667, *p* = 0.002) and 1.634 times (95% CI: 1.135–2.354, *p* = 0.008) more likely to feel neutral than those who required full assistance, respectively. Those who could go out alone were 1.395 times (95% CI: 1.076–1.808, *p* = 0.012) more likely to feel neutral than those who could not. Those who engaged in paid work were 2.311 times (95% CI: 1.635–3.267, *p* < 0.001) more likely to feel neutral than those who did not. Those who participated in social activities were 1.661 times (95% CI: 1.298–2.126, *p* < 0.001) more likely to feel neutral than those who did not.

Sex, age, education level, marital status, degree of stress, presence of sadness and despair, presence of suicidal thoughts, perceived body shape, exercise participation, presence of chronic disease, ability to go out alone, engagement in paid work, difficulty in communication, degree of discrimination, and participation in social activities were associated with feeling happy among individuals with severe disability. Those who were male were 0.692 times (95% CI: 0.532–0.901, *p* = 0.006) less likely to feel happy than those who were female. Those who were aged 20–29 were 2.309 times (95% CI: 1.062–5.021, *p* = 0.035) more likely to feel happy than those aged 80 years or older. Those who had elementary school or lower education levels, those who had middle school education level, and those who had high school education level were 0.349 times (95% CI: 0.233–0.524, *p* <0.001), 0.469 times (95% CI: 0.305–0.721, *p* = 0.001), and 0.696 times (95% CI: 0.495–0.980, *p* = 0.038) less likely to feel happy than those who had college or higher education levels, respectively. Those who were married were 3.060 times (95% CI: 2.058–4.551, *p* <0.001) more likely to feel happy than those who were unmarried.

Those who felt stressed every moment, those who felt stressed a lot, and those who a little felt stressed were 0.108 times (95% CI: 0.059–0.198, *p* < 0.001), 0.168 times (95% CI: 0.114–0.246, *p* < 0.001), and 0.549 times (95% CI: 0.381–0.789, *p* = 0.001) less likely to feel happy than those who almost never felt stressed, respectively. Those who experienced sadness and despair were 0.385 times (95% CI: 0.255–0.580, *p* < 0.001) less likely to feel happy than those who did not. Those who experienced suicidal thoughts were 0.328 times (95% CI: 0.193–0.558, *p* < 0.001) less likely to feel happy than those who did not. Those who perceived their body shape as very thin were 0.400 times (95% CI: 0.209–0.767, *p* = 0.006) less likely to feel happy than those who perceived their body shape as very obese. Those who exercised were 3.352 times (95% CI: 2.576–4.362, *p* < 0.001) more likely to feel happy than those who did not. Those who suffered from a chronic disease were 0.515 times (95% CI: 0.343–0.774, *p* = 0.001) less likely to feel happy than those who did not. Those who could go out alone were 1.713 times (95% CI: 1.226–2.393, *p* = 0.002) more likely to feel happy than those who could not.

Those who engaged in paid work were 4.161 times (95% CI: 2.870–6.031, *p* < 0.001) more likely to feel happy than those who did not. Those who experienced difficulty in communication were 0.743 times (95% CI: 0.561–0.984, *p* = 0.038) less likely to feel happy than those who did not. Those who always experienced discrimination, those who sometimes experienced discrimination, and those who rarely experienced discrimination were 0.230 times (95% CI: 0.126–0.420, *p* < 0.001), 0.390 times (95% CI: 0.251–0.606, *p* < 0.001), and 0.616 times (95% CI: 0.401–0.948, *p* = 0.028) less likely to feel happy than those who did not experience discrimination at all, respectively. Those who participated in social activities were 2.764 times (95% CI: 2.088–3.660, *p* < 0.001) more likely to feel happy than those who did not.

These results suggested that among individuals with severe disability, males are less happy than females. Those who are aged 20–29 are happier than those aged 80 years or older. Those who have elementary school or lower education levels are less happy than those who have college or higher education levels. Those who are married are happier than those who are unmarried. Those who experience stress, sadness and despair, suicidal thoughts, suicide attempts, difficulty in communication, or discrimination are less happy than those who do not. Those who perceive their body as very thin are less happy than those who perceive it as very obese. Those who suffer from a chronic disease are less happy than those who do not. In contrast, those who exercise, go out alone, engage in paid work, or participate in social activities are happier than those who do not.

## 4. Discussion

Being married, participating in exercise, being able to go out alone, engaging in paid work, and participating in social activities were associated with increased happiness among individuals with mild disability and those with severe disability. Being male, having lower levels of education, experiencing stress, sadness and despair, or suicidal thoughts, and feeling discriminated against were associated with reduced happiness among individuals with mild disability and those with severe disability.

Males with disabilities were found to be less happy than females with disabilities. Emerson et al. [21] studied the personal well-being of individuals with and without disabilities in the United Kingdom and found that being male or young and not having a partner reduced their well-being. Additionally, Emerson et al. [22] reported that women with disabilities are 14–15% less likely to be happy than individuals without disabilities, whereas men with disabilities are 15–17% less likely [22]. Men with disabilities are less happy than women with disabilities because men with disabilities tend to be more reluctant to receive care and support from strangers than women with disabilities, which seems to have a negative impact on their life satisfaction and happiness [23,24]. This makes it necessary to find ways to increase the happiness of men with disabilities.

Individuals with disabilities with lower education levels were found to be less happy than individuals with disabilities with higher education levels. Cuñado et al. [25] found that people with higher education levels have higher income levels and a higher probability of being employed; thus, they report higher levels of happiness. In the Republic of Korea, students strive to study at a high-ranking university because it helps them secure a good job, high social status, and a good partner [26]. In other words, in the Republic of Korea, one’s education level directly and indirectly affects many elements of one’s life. Despite revisions to the Disability Discrimination Act, social awareness and support systems for individuals with disabilities remain insufficient. Consequently, these individuals face difficulty in securing employment and experience discrimination [27,28]. Thus, it can be inferred that in the Republic of Korea, individuals with disabilities with lower education levels face challenges in securing employment, social status, and marriage, which lowers their happiness.

Married individuals with disabilities were found to be happier than those who are unmarried. Studies have shown that married individuals are healthier, happier, and live longer than those who are divorced, widowed, or unmarried [29,30]. In this context, it can be concluded that being married is associated with feeling happy among individuals with disabilities [30]. This may be because marriage tends to reduce loneliness and depression and increase one’s engagement with their partner, thus increasing happiness levels [31]. Despite the positive impact of marriage on the happiness of individuals with disabilities, these individuals are more likely to remain single than to get married for practical reasons [32]. This makes it important to implement welfare policies that encourage these individuals to get married and support their married life.

Regarding mental health factors, experiencing stress, sadness and despair, or suicidal thoughts were associated with reduced happiness among individuals with disabilities. Kye et al. [33] found that low levels of stress determine health-related happiness. Choi et al. [34] reported that various factors, including socioeconomic, psychological, and health-related factors, affect happiness and suicide and that unhappiness and suicidal thoughts are closely related. Individuals with disabilities experience stress, sadness and despair, and suicidal behaviors because of physical health problems, social prejudice, economic situations, and loneliness [11,12,13,14,15]. These mental problems cause them to suffer from depression, anxiety, and psychological distress [11]. This might be why individuals with disabilities who experience stress, sadness and despair, or suicidal thoughts tend to be unhappy. Support is needed to improve the mental health of these individuals and increase their happiness.

Regarding physical health factors, participating in exercise and being able to go out alone were associated with increased happiness among individuals with disabilities. Studies have reported that physical activity has a significant effect on happiness [16]. For individuals with disabilities, physical activity contributes to functional independence, improves physical condition, performance ability, and physical ability; helps prevent and correct deformities and postural defects; reduces stress; and increases self-confidence, emotional state, relationships with others, enjoyment, and interest [35,36,37,38]. These positive effects of exercise participation seem to increase the happiness levels of individuals with disabilities. Overall, participation in physical activity can be a way to increase the happiness of individuals with disabilities. However, only 65.8% of the respondents in this study participated in physical activity. This highlights the need to increase support for individuals with disabilities so that they can increase exercise participation and enhance their physical health as well as happiness. However, being able to go out alone can be an indicator that one can live independently [39,40,41,42]. Several studies have reported that going out alone positively affects independent living, well-being, and health-related quality of life [39,40,41,42]. Therefore, it seems that individuals with disabilities who go out alone feel happy because they live an independent life that does not require the help of others. This makes it vital to provide welfare services that allow individuals with disabilities to go out alone.

Regarding social health factors, engaging in paid work and participating in social activities were associated with increased happiness among individuals with disabilities, whereas experiencing discrimination was associated with decreased happiness. Having regular positive interactions with family, friends, and coworkers has a positive impact on people’s health. Pagán et al. [18] reported that having good relationships with colleagues, superiors, and work increases the job satisfaction and happiness of individuals with disabilities [18]. Liu et al. [19] emphasized the need for community life for individuals with disabilities, as social interactions positively affect their happiness. In other words, continuous interaction with people, which can be achieved by engaging in paid work and participating in social activities, tends to have a positive effect on the health, happiness, and consequently, the well-being of individuals with disabilities. However, experiencing discrimination, social isolation, or lack of close social ties hamper health. Individuals with disabilities often experience social discrimination, financial difficulties, and restricted social participation than those without disabilities [27,28]. Discrimination and prejudice against these individuals are likely to isolate and discourage them, thus creating an adverse impact on their health [28]. Therefore, it is necessary to create a social environment where individuals with disabilities can interact with people and do what they want without facing discrimination.

Younger age (20–29 years) predicted higher happiness levels among individuals with severe disabilities but not among those with moderate disabilities. This result can be explained in two ways. First, the degree of acceptance of disability varies depending on the degree of disability. It has been reported that a greater acceptance of their disability is linked to better mental health among individuals with disabilities [43]. Disability acceptance varies depending on the individual, but it is affected by the degree of disability. According to Song [44], acceptance of disability may increase with the period of disability; furthermore, the acceptance of disability is reported to be higher with a greater degree of disability. As such, the degree of acceptance of disability varies depending on the degree of disability, and this attitude seems to affect happiness among individuals with disabilities. Second, social support in Korea varies depending on the degree of disability. Although the disability rating system has been dualized according to the degree of disability, the provided economic support and social welfare benefits are greater with a greater degree of disability; thus, the rating system has not been completely abolished [45]. People with disabilities often complain of economic difficulties, and it is possible that their sense of happiness is affected by welfare benefits provided by the government.

This study has some limitations. First, there are various types of disabilities, such as visual impairment, hearing impairment, and developmental disability; however, this study did not determine the type of disability. Future research should consider the type of disability and should explore health factors that affect happiness by considering the level and type of disability. Second, this study targeted Korean individuals with disabilities aged 20 or older. However, 70% of the respondents were in their 50s or older. Therefore, it can be interpreted that the results were majorly influenced by the data of middle-aged and older individuals with disabilities. Third, because this was a cross-sectional study, we could not determine causality. We could only assess the relationship between health-related factors and the happiness of Korean adults with disabilities based on their disability level. Fourth, this study focused on Korean adults with disabilities. Owing to the different socio-economic and cultural background in Korea, the findings of this study cannot be generalized to people with disabilities worldwide. Fifth, this research is intended to ensure the success of a future large-scale study that will examine the research question closely. Thus, the validity or reliability of the survey instrument was not evaluated. Sixth, answers about happiness may be influenced by the research participants’ situations, such as health status and mood, and the researcher reviewed the participants’ answers on happiness. In this process, the research participants’ attitudes toward happiness may have been modified. Nevertheless, the results of this study are expected to help establish disability-degree-based welfare policies that enhance the happiness of adults with disabilities. Moreover, because this study used a large and nationally representative sample, our findings should be generalizable to other research settings.

## 5. Conclusions

Being male, having lower education levels, and experiencing stress, sadness and despair, suicidal thoughts, or discrimination are associated with reduced happiness among individuals with moderate disability. In contrast, being married, exercising, being able to go out alone, engaging in paid work, and participating in social activities are associated with increased happiness. Being male, being 20–29 years old, having lower education levels, experiencing stress, sadness and despair, suicidal thoughts, suicide attempts, difficulty in communication, or discrimination, perceiving body shape as very thin, and suffering from a chronic disease are associated with reduced happiness among individuals with severe disability. In contrast, being married, exercising, being able to go out alone, engaging in paid work, and participating in social activities are associated with increased happiness.

In terms of demographic factors, males with disabilities are less happy than females with disabilities. Those with lower levels of education are less happy than those with higher levels of education. However, married individuals with disabilities are happier than those who are unmarried. In terms of mental health factors, individuals with disabilities who experience stress, sadness and despair, or suicidal thoughts are less happy than those who do not. In terms of physical health factors, individuals with disabilities who participate in exercise and go out alone are happier than those who do not. In terms of social health factors, individuals with disabilities who engage in paid work and participate in social activities are happier than those who do not. However, individuals with disabilities who experience discrimination are less happy than those who do not experience discrimination. In other words, the factors affecting the happiness of Korean adults with disabilities differ depending on the degree of disability. Health policies, plans, and support measures should be formulated based on the degree of disability to increase the happiness of individuals with disabilities. For example, as a part of these measures, it will be possible to establish policies to support daily physical activities according to the degree of disability as well as health services to support physical activity.

## Figures and Tables

**Table 1:** Characteristics of the respondents (*n* = 7581).

Variables			*n* (%)
Happiness		Unhappy	1068 (14.1%)
		Neutral	3835 (50.6%)
		Happy	2678 (35.3%)
Demographic factors	Sex	Male	4574 (60.3%)
		Female	3007 (39.7%)
	Age	20–29 years	341 (4.5%)
		30–39 years	334 (4.4%)
		40–49 years	726 (9.6%)
		50–59 years	1261 (16.6%)
		60–69 years	1929 (25.4%)
		70–79 years	1669 (22.0%)
		80 years or older	1321 (17.5%)
	Disability level	Moderate disability	4001 (52.8%)
		Severe disability	3580 (47.2%)
	Education level	Elementary school or below	2380 (31.4%)
		Middle school	1191 (15.7%)
		High school	2450 (32.3%)
		College or higher	1560 (20.6%)
	Marital status	Married	3784 (49.9%)
		Widowed/divorced/separated/other (single mother/single father, etc.)	2278 (30.1%)
		Unmarried	1519 (20.0%)
Mental health factors	Degree of stress	Every moment	464 (6.1%)
		A lot	2076 (27.4%)
		A little	3414 (45.0%)
		Almost never	1627 (21.5%)
	Presence of sadness and despair	Yes	1042 (13.7%)
		No	6539 (86.3%)
	Presence of suicidal thoughts	Yes	718 (9.5%)
		No	6863 (90.5%)
	Engagement in suicide attempts	Yes	63 (0.8%)
		No	7518 (99.2%)
	Experience of psychological counseling	Yes	442 (5.8%)
		No	7139 (94.2%)
Physical health factors	Perceived body shape	Very thin	447 (5.9%)
		Slightly thin	1410 (18.5%)
		Normal	3560 (47.0%)
		Slightly obese	1780 (23.5%)
		Very obese	384 (5.1%)
	Exercise participation	Yes	4991 (65.8%)
		No	2590 (34.2%)
	Presence of chronic disease	Yes	6489 (85.6%)
		No	1092 (14.4%)
	Requirement of walking assistance	No assistance required	5769 (76.1%)
		Some assistance required	1135 (15.0%)
		Considerable assistance required	371 (4.9%)
		Full assistance required	306 (4.0%)
	Ability to go out alone	Yes	6096 (80.4%)
		No	1485 (19.6%)
Social health factors	Engagement in paid work	Yes	2502 (33.0%)
		No	5079 (67.0%)
	Difficulty in communication	Yes	1862 (24.6%)
		No	5719 (75.4%)
	Utilization of health services	Yes	7464 (98.5%)
		No	117 (1.5%)
	Degree of discrimination	Always	336 (4.5%)
		Sometimes	2163 (28.5%)
		Rarely	3745 (49.4%)
		Not at all	1337 (17.6%)
	Participation in social activities	Yes	3821 (50.4%)
		No	3760 (49.6%)

**Table 2:** Factors that influence the happiness of individuals with moderate disability.

Variables			Neutral		Happy	
			OR (95% CI)	*p*	OR (95% CI)	*p*
Demographic factors	Sex	Male	0.589 (0.444–0.780)	<0.001 ***	0.397 (0.291–0.542)	<0.001 ***
		Female	Reference		Reference	
	Age	20–29 years	1.992 (0.436–9.098)	0.374	3.259 (0.649–16.362)	0.151
		30–39 years	5.517 (0.626–48.630)	0.124	6.163 (0.676–56.191)	0.107
		40–49 years	0.539 (0.284–1.023)	0.059	0.668 (0.333–1.340)	0.256
		50–59 years	0.686 (0.416–1.131)	0.139	0.720 (0.416–1.244)	0.239
		60–69 years	0.710 (0.481–1.048)	0.084	0.651 (0.423–1.002)	0.051
		70–79 years	0.919 (0.642–1.318)	0.647	0.807 (0.541–1.205)	0.295
		80 years or older	Reference		Reference	
	Education level	Elementary school or below	0.991 (0.649–1.513)	0.967	0.364 (0.231–0.575)	<0.001 ***
		Middle school	1.580 (0.989–2.524)	0.056	0.635 (0.384–1.050)	0.077
		High school	1.388 (0.935–2.061)	0.104	0.775 (0.511–1.176)	0.231
		College or higher	Reference		Reference	
	Marital status	Married	2.014 (1.266–3.202)	0.003 **	6.006 (3.549–10.162)	<0.001 ***
		Widowed/divorced/separated/other (single mother/single father, etc.)	0.988 (0.615–1.587)	0.960	1.117 (0.645–1.934)	0.694
		Unmarried	Reference		Reference	
Mental health factors	Degree of stress	Every moment	0.200 (0.115–0.348)	<0.001 ***	0.063 (0.032–0.122)	<0.001 ***
		A lot	0.368 (0.237–0.571)	<0.001 ***	0.092 (0.058–0.148)	<0.001 ***
		A little	0.709 (0.458–1.099)	0.124	0.395 (0.252–0.619)	<0.001 ***
		Almost never	Reference		Reference	
	Presence of sadness and despair	Yes	0.587 (0.426–0.809)	0.001 **	0.429 (0.280–0.658)	<0.001 ***
		No	Reference		Reference	
	Presence of suicidal thoughts	Yes	0.555 (0.392–0.785)	0.001 **	0.279 (0.167–0.468)	<0.001 ***
		No	Reference		Reference	
	Engagement in suicide attempts	Yes	0.400 (0.140–1.138)	0.086	1.171 (0.301–4.561)	0.820
		No	Reference		Reference	
	Experience of psychological counseling	Yes	1.127 (0.680–1.867)	0.643	0.906 (0.480–1.711)	0.760
		No	Reference		Reference	
Physical health factors	Perceived body shape	Very thin	0.863 (0.435–1.712)	0.674	1.130 (0.501–2.550)	0.769
		Slightly thin	0.784 (0.446–1.375)	0.396	0.785 (0.399–1.542)	0.481
		Normal	1.222 (0.713–2.096)	0.465	1.467 (0.770–2.794)	0.244
		Slightly obese	1.062 (0.605–1.864)	0.835	1.442 (0.740–2.811)	0.282
		Very obese	Reference		Reference	
	Exercise participation	Yes	1.654 (1.274–2.147)	<0.001 ***	2.369 (1.765–3.180)	<0.001 ***
		No	Reference		Reference	
	Presence of chronic disease	Yes	0.757 (0.436–1.314)	0.322	0.655 (0.371–1.157)	0.145
		No	Reference		Reference	
	Requirement of walking assistance	No assistance required	1.590 (0.700–3.611)	0.268	2.251 (0.656–7.721)	0.197
		Some assistance required	1.649 (0.741–3.668)	0.220	2.036 (0.602–6.886)	0.253
		Considerable assistance required	1.225 (0.506–2.968)	0.653	1.860 (0.490–7.068)	0.362
		Full assistance required	Reference		Reference	
	Ability to go out alone	Yes	1.538 (1.038–2.279)	0.032 *	1.826 (1.116–2.988)	0.017 *
		No	Reference		Reference	
Social health factors	Engagement in paid work	Yes	2.121 (1.507–2.986)	<0.001 ***	3.726 (2.598–5.345)	<0.001 ***
		No	Reference		Reference	
	Difficulty in communication	Yes	1.177 (0.841–1.647)	0.343	0.977 (0.664–1.439)	0.906
		No	Reference		Reference	
	Utilization of health services	Yes	2.222 (0.898–5.499)	0.084	2.720 (0.979–7.560)	0.055
		No	Reference			
	Degree of discrimination	Always	0.516 (0.259–1.027)	0.059	0.125 (0.048–0.323)	<0.001 ***
		Sometimes	0.636 (0.421–0.960)	0.031 *	0.259 (0.166–0.404)	<0.001 ***
		Rarely	0.860 (0.587–1.260)	0.439	0.483 (0.325–0.718)	<0.001 ***
		Not at all	Reference		Reference	
	Participation in social activities	Yes	1.349 (1.035–1.758)	0.027 *	2.246 (1.680–3.002)	<0.001 ***
		No	Reference		Reference	

* *p* < 0.05, ** *p* < 0.01, *** *p* < 0.001; assessed through multivariate logistic regression analysis. OR: odds ratio; CI: confidence interval.

**Table 3:** Factors that influence the happiness of individuals with severe disability.

Variables			Neutral		Happy	
			OR (95% CI)	*p*	OR (95% CI)	*p*
Demographic factors	Sex	Male	0.824 (0.663–1.023)	0.079	0.692 (0.532–0.901)	0.006 **
		Female	Reference		Reference	
	Age	20–29 years	1.340 (0.696–2.581)	0.381	2.309 (1.062–5.021)	0.035 *
		30–39 years	0.917 (0.498–1.689)	0.781	1.264 (0.601–2.658)	0.537
		40–49 years	0.726 (0.437–1.206)	0.216	0.885 (0.477–1.644)	0.700
		50–59 years	0.693 (0.461–1.041)	0.077	0.624 (0.373–1.042)	0.071
		60–69 years	0.880 (0.617–1.254)	0.480	0.824 (0.525–1.292)	0.398
		70–79 years	1.104 (0.781–1.561)	0.575	0.846 (0.540–1.327)	0.467
		80 years or older	Reference		Reference	
	Education level	Elementary school or below	0.704 (0.501–0.989)	0.043 *	0.349 (0.233–0.524)	<0.001 ***
		Middle school	0.819 (0.569–1.177)	0.281	0.469 (0.305–0.721)	0.001 **
		High school	0.919 (0.679–1.243)	0.583	0.696 (0.495–0.980)	0.038 *
		College or higher	Reference		Reference	
	Marital status	Married	1.242 (0.896–1.721)	0.194	3.060 (2.058–4.551)	<0.001 ***
		Widowed/divorced/separated/other (single mother/single father, etc.)	0.855 (0.609–1.201)	0.365	0.768 (0.496–1.188)	0.236
		Unmarried	Reference		Reference	
Mental health factors	Degree of stress	Every moment	0.296 (0.194–0.452)	<0.001 ***	0.108 (0.059–0.198)	<0.001 ***
		A lot	0.404 (0.290–0.562)	<0.001 ***	0.168 (0.114–0.246)	<0.001 ***
		A little	0.820 (0.588–1.144)	0.242	0.549 (0.381–0.789)	0.001 **
		Almost nothing	Reference		Reference	
	Presence of sadness and despair	Yes	0.560 (0.425–0.737)	<0.001 ***	0.385 (0.255–0.580)	<0.001 ***
		No	Reference		Reference	
	Presence of suicidal thoughts	Yes	0.776 (0.570–1.057)	0.107	0.328 (0.193–0.558)	<0.001 ***
		No	Reference		Reference	
	Engagement in suicide attempts	Yes	0.242 (0.106–0.551)	0.001 **	0.288 (0.055–1.521)	0.143
		No	Reference		Reference	
	Experience of psychological counseling	Yes	1.104 (0.775–1.571)	0.584	0.958 (0.611–1.502)	0.851
		No	Reference		Reference	
Physical health factors	Perceived body shape	Very thin	0.730 (0.438–1.215)	0.226	0.400 (0.209–0.767)	0.006 **
		Slightly thin	1.005 (0.639–1.581)	0.982	0.726 (0.424–1.244)	0.244
		Normal	1.072 (0.701–1.639)	0.750	0.877 (0.532–1.445)	0.607
		Slightly obese	1.098 (0.710–1.698)	0.674	0.793 (0.474–1.327)	0.377
		Very obese	Reference		Reference	
	Exercise participation	Yes	1.974 (1.572–2.412)	<0.001 ***	3.352 (2.576–4.362)	<0.001 ***
		No	Reference		Reference	
	Presence of chronic disease	Yes	0.721 (0.500–1.039)	0.079	0.515 (0.343–0.774)	0.001 **
		No	Reference		Reference	
	Requirement of walking assistance	No assistance required	1.634 (1.135–2.354)	0.008 **	1.258 (0.749–2.110)	0.385
		Some assistance required	1.826 (1.251–2.667)	0.002 **	1.471 (0.858–2.522)	0.161
		Considerable assistance required	1.148 (0.759–1.736)	0.514	0.827 (0.445–1.537)	0.549
		Full assistance required	Reference		Reference	
	Ability to go out alone	Yes	1.395 (1.076–1.808)	0.012 *	1.713 (1.226–2.393)	0.002 **
		No	Reference		Reference	
Social health factors	Engagement in paid work	Yes	2.311 (1.635–3.267)	<0.001 ***	4.161 (2.870–6.031)	<0.001 ***
		No	Reference		Reference	
	Difficulty in communication	Yes	0.824 (0.656–1.035)	0.095	0.743 (0.561–0.984)	0.038 *
		No	Reference		Reference	
	Utilization of health Services	Yes	0.591 (0.241–1.449)	0.251	0.888 (0.311–2.535)	0.825
		No	Reference		Reference	
	Degree of discrimination	Always	0.693 (0.426–1.128)	0.140	0.230 (0.126–0.420)	<0.001 ***
		Sometimes	0.887 (0.598–1.317)	0.553	0.390 (0.251–0.606)	<0.001 ***
		Rarely	1.115 (0.753–1.651)	0.586	0.616 (0.401–0.948)	0.028 *
		Not at all	Reference		Reference	
	Participation in social activities	Yes	1.661 (1.298–2.126)	<0.001 ***	2.764 (2.088–3.660)	<0.001 ***
		No	Reference		Reference	

* *p* < 0.05, ** *p* < 0.01, *** *p* <0.001; assessed through multivariate logistic regression analysis. OR: odd ratio; CI: confidence interval.

## Data Availability

Publicly available datasets were analyzed in this study. These data can be found at the following link: https://data.kihasa.re.kr/kihasa/kor/contents/ContentsList.html (accessed on 5 March 2025).
